# Circulating exosomal microRNA‐96 promotes cell proliferation, migration and drug resistance by targeting LMO7

**DOI:** 10.1111/jcmm.13056

**Published:** 2016-12-27

**Authors:** Hao Wu, Jingcheng Zhou, Shanshan Mei, Da Wu, Zhimin Mu, Baokun Chen, Yuancai Xie, Yiwang Ye, Jixian Liu

**Affiliations:** ^1^ Department of Thoracic SurgeryPeking University Shenzhen HospitalShenzhenGuangdong ProvinceChina

**Keywords:** lung cancer, miR‐96, exosome, LMO7, drug resistance

## Abstract

Detection and treatment of lung cancer still remain a clinical challenge. This study aims to validate exosomal microRNA‐96 (miR‐96) as a serum biomarker for lung cancer and understand the underlying mechanism in lung cancer progression. MiR‐96 expressions in normal and lung cancer patients were characterized by qPCR analysis. Changes in cell viability, migration and cisplatin resistance were monitored after incubation with isolated miR‐96‐containing exosomes, anti‐miR‐96 and anti‐miR negative control (anti‐miR‐NC) transfections. Dual‐luciferase reporter assay was used to study interaction between miR‐96 and LIM‐domain only protein 7 (LMO7). Changes induced by miR‐96 transfection and LMO7 overexpression were also evaluated. MiR‐96 expression was positively correlated with high‐grade and metastatic lung cancers. While anti‐miR‐96 transfection exhibited a tumour‐suppressing function, exosomes isolated from H1299 enhanced cell viability, migration and cisplatin resistance. Potential miR‐96 binding sites were found within the 3′‐UTR of wild‐type *LMO7* gene, but not of mutant LMO7 gene. LMO7 expression was inversely correlated with lung cancer grades, and LMO7 overexpression reversed promoting effect of miR‐96. We have identified exosomal miR‐96 as a serum biomarker of malignant lung cancer. MiR‐96 promotes lung cancer progression by targeting LMO7. The miR‐96‐LMO7 axis may be a therapeutic target for lung cancer patients, and new diagnostic or therapeutic strategies could be developed by targeting the miR‐96‐LMO7 axis.

## Introduction

Lung cancer is one of the most common and deadly cancers worldwide. More people die from lung cancer than from prostate and breast cancers combined 1, 2. Prognosis of lung cancer remains poor due to its high probability of metastasis, recurrence and drug resistance 3, 4. Poor outcome and frequent relapses associated with lung cancer urgently demand development of new screening and early biomarkers for accurate and non‐invasive detection of lung cancer metastasis and recurrence.

MiRNAs are frequently dysregulated in lung cancer and are implicated in lung cancer growth, invasion, recurrence and metastasis 5. MiRNAs are non‐coding small RNAs, approximately 20–25 nucleotide long, that post‐transcriptionally regulate a broad spectrum of mRNAs intracellularly. Thus, miRNAs are capable of regulating a number of physiological processes 6, 7. Even though miRNAs function primarily intracellularly, emerging evidence suggested that miRNAs could be secreted into extracellular compartments, transferred between cells, which consequently regulate recipient cell physiology. During these processes, miRNAs are packaged in lipoproteins, or vehicles called ‘exosomes’, as messengers to communicate between cells and extracellular microenvironment 8.

Exosomally transferred miRNAs have been regarded as novel regulators in cancer progression. Cancer cells that receive exosomal miRNAs undergo physiological changes that either promote or repress their proliferation and migration abilities, leading to tumour cell invasion into the blood vessel, survival in circulation and eventually dissemination in secondary organs. These induced changes may also endow cells resistance to chemotherapy and radiotherapy. Among all miRNAs investigated, miR‐96 has drawn much attention and has been considered as a cancer promoter in prostate cancer 9, bladder cancer 10 and hepatoma cancer 11. Recently, an elevated level of miR‐96 has also been reported in non‐small cell lung cancer (NSCLC) 12, 13. Despite the important role of miR‐96 in lung cancer, the detailed mechanism of miR‐96 action remains unclear.

LMO7, a fibrous actin‐binding protein that is widely expressed in adult tissues, is a member of PDZ and LIM domain‐containing protein family, which function as protein–protein recognition modules 14. In normal tissue, LMO7 is considered as a molecule that aids in the formation and maintenance of epithelial architecture *via* remodelling of actin cytoskeleton. In cancer tissue, increased expression of LMO7 has been reported in colorectal, breast, liver, lung pancreas, stomach and prostate cancers, suggesting that an important role of LMO7 in cytoskeletal reorganization during carcinogenesis 15. In lung cancer, LMO7 functions as a tumour suppressor and its deficiency confers a genetic predisposition to lung cancer 15. However, mechanism regulating LMO7 expression in lung cancer is still yet to be understood.

Herein, using clinical samples from lung cancer patients, we found that miR‐96 is up‐regulated in patients with lung cancer, especially with high‐grade lung cancers. Exosomal miR‐96 is also positively correlated with lung cancer risk. Transfection with anti‐miR‐96 compromises the tumour‐promoting function of miR‐96. We also confirmed that LMO7 is down‐regulated in lung cancer. LMO7 is a target of miR‐96, and overexpression of LMO7 could reverse the promoting effect of miR‐96 in lung cancer.

## Materials and methods

### Cell culture and viability assay

All cell lines used in this study, including BEAS‐2B, A549, PC9 and H1299, were purchased from American Type Culture Collection (ATCC, Manassas, VA, USA). Cells were cultured in RPMI medium containing 10% foetal bovine serum (FBS). For viability assay, cells were firstly seeded in 96‐well plates; 10 μl of Cell Count Kit‐8 (CCK‐8; Sigma‐Aldrich, St. Louis, MO, USA) solution was added into each well. Cells were then incubated in CCK‐8 solution for 4 hrs, and absorption value at 450 nm was measured by a plater reader.

### Migration assay

Scratch wound analysis was carried out by using 10‐μl pipette tip to enforce wound areas on a plate with over 80% confluence. Phase contrast images of the gaps were taken at a time interval of 4 hrs after gaps were made. Gap areas were presented as ratios of initial gap area and quantified by ImageJ. Transwell Matrigel invasion assay was also performed in 24‐well transwell units (Corning, New York, NY, USA). 10^5^ cells were seeded in the upper chamber, which were coated with Matrigel; 500 μl RPMI was applied in the lower chamber. Invading cells in the bottom chamber were fixed and analysed by measuring absorbance at 570 nm after a 24‐hrs incubation.

### Isolation of exosomes

Isolation of exosomes from the serum of patients was performed with the ExoQuick‐TC method (System Biosciences, Palo Alto, CA, USA) according to the manufacturer's protocols. ExoQuick‐TC was also used to obtain exosomes from medium of H1299. After cell cultures reached 80% confluency (about 5 × 10^6^ cells), cells were washed with PBS and incubated with freshly prepared complete medium containing exosome‐free FBS for 48 hrs. The conditioned medium was collected and centrifuged at 2000 × *g* for 20 min., followed by filtration through a 0.22‐μm filter to remove all cell debris; 10 ml of supernatant was mixed with 2 ml of ExoQuick precipitation solution and incubated overnight at 4°C, followed by centrifugation at 500 × *g* for 30 min. to precipitate exosomes. Pellet containing exosomes was resuspended in 100 μl phosphate‐buffered saline (PBS) and washed by centrifugation. Exosomes from the conditioned medium of A549 cells were isolated in the similar way, except that H1299‐derived exosomes, which were used to treat A549 cell, were depleted by replacing the medium at 48 hrs prior to exosomes isolation. To evaluate the efficiency of exosome isolation using ExoQuick precipitation, CD63 and heat‐shock protein 70 (HSP70) levels in isolated exosomes were evaluated with Western blot by loading equivalent amount of proteins. CD63 and HSP70 levels in exosomes isolated from ultracentrifugation used for comparison. We showed that exosomes isolated using ExoQuick protocols demonstrated similar CD63 and HSP expressions with those isolated by ultracentrifugation (Fig. S1).

### Quantitative reverse‐transcription PCR

Tissues for miR‐96 and LMO7 analysis were collected from Peking University Shenzhen Hospital. Total RNA from tissue or cells were extracted using TRIzol reagent (Invitrogen, Pleasanton, CA, USA), and genomic DNA were removed using ThurboDNase kit (Amibion, Waltham, MA, USA) and quantified by NanoDrop. Synthesis of cDNA was performed with PrimeScriptRT reagent KIT (Takara Bio, Tokyo, Japan) according to the manufacturer's instruction. Reverse‐transcription reaction and quantitative PCR (qPCR) assay were performed with All‐in‐One™ miRNA qRT‐PCR Detection Kit (AOMD‐Q020; GeneCopoeia, Rockville, MD, USA) on a 7900HT system supplied with analytical software (Applied Biosystems, Waltham, MA, USA). U6 was selected as endogenous reference. The primers and probes for miR‐96 and U6 were obtained from GeneCopoeia. Quantitative reverse‐transcriptase (qRT) PCR GAPDH and LMO7 primers were the following: LMO7 forward primer, 5′‐ GTCTACAGTTCCGTCAAGAAGG‐3′; LMO7 reverse primer, 5′‐ TCTGAAGGATAAGTTGCTCCCT‐3′; GAPDH forward primer, 5′‐CCACCCATGGCAAATTCCATGGCA‐3′; GAPDH reverse primer, 5′‐TCTAGACGGCAGGTCAGGTCCACC ‐3′. GAPDH or U6 levels were used as an internal control, and fold changes were calculated by relative quantification (2^−ΔΔCt^).

### Western Blot

For Western blot analysis, tissue samples were homogenized. Pierce BCA Protein Assay Kit (Thermo Fisher, Waltham, MA, USA) was used to quantify protein concentration in tissue lysates. Electrophoresis was performed with Mini‐PROTEAN tetra Cell system (Bio‐Rad, Hercules, CA, USA) using precast gels and blotted with nitrocellulose membranes. Rabbit polyclonal antibodies specific to LMO7 were purchased from Abcam (ab86069; Cambridge, MA, USA) and used to incubate with the membranes for 1 hr at room temperature. After extensive washing, horseradish peroxidase (HRP) conjugated goat anti‐rabbit secondary antibody (ab6721; Abcam) was subsequently applied to the membranes. The membranes were then reacted with ECL Western blot substrate kit (Pierce, Waltham, MA, USA) before exposure.

### Lentivirus packaging

To stably express anti‐miR‐96, anti‐miR‐NC, miR‐96 and LMO7 in A549 cells, the lentiviruses carrying miR‐96, negative control (miR‐NC), or LMO7 cDNA were packaged using the lentiviral packaging kit (Thermo Fisher Scientific) following the manufacturer's manual. Lentivirus was packaged in A549 cells and secreted into the medium. A549 cells were infected by lentivirus carrying miR‐96, miR‐NC, or LMO7 cDNA with the presence of polybrene (Sigma‐Aldrich) and selected by puromycin for 2 weeks to obtain stable cell lines.

### Dual‐luciferase reporter assay

Wild‐type and mutant LMO7 genes were subcloned into pGL3 Basic luciferase reporter plasmid (Promega, Madison, WI, USA). FuGENE 6 (Boehringer, Ingelheim am Rhein, Germany) was used for transfections. Reporter gene assays were performed with the Dual‐luciferase reporter assay system (Promega); 50 ng pRL‐TK (Promega) Renilla luciferase was cotransfected in each sample as an internal control for transfection efficiency.

### Statistical analysis

One‐ or two‐way anova followed by a Tukey's *post hoc* test was performed to evaluate the significance of differences. Spearman's correlation was used to determine the relationship between two factors. Differences were considered statistically significant at *P* < 0.05.

## Results

### MiR‐96 is a biomarker for lung cancer

**Figure 1:**
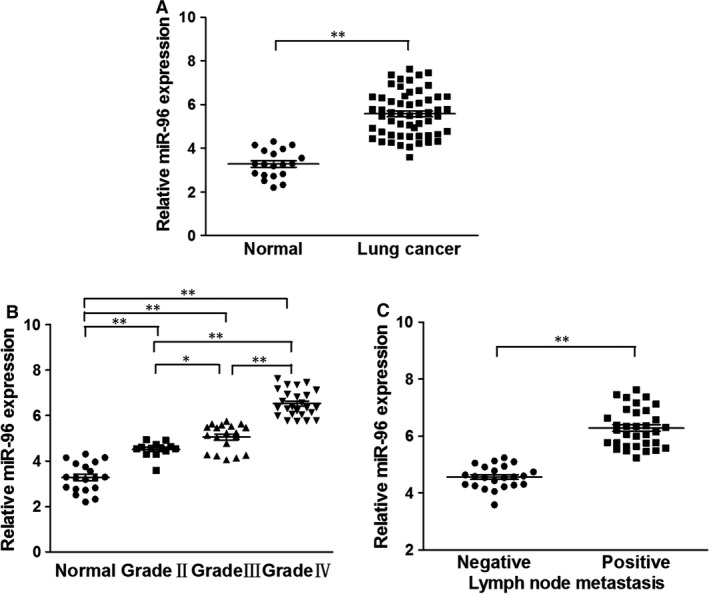
miR‐96 levels are up‐regulated in human lung cancer tissues and correlated to metastasis. (**A**) Relative miR‐96 expression levels were analysed by qRT‐PCR in normal (*n* = 19) and lung cancer tissues (*n* = 56). U6 RNA levels were used as an internal control. (**B**) Relative expression levels of miR‐96 in different stages of cancer tissues. (**C**) Relative expression levels of miR‐96 in different types of lymph node metastasis. Data were presented as the means ± S.D. Experiments were repeated in triplicate. *P < 0.05, **P < 0.01.

To validate high miR‐96 level as a biomarker for lung cancer, we first collected lung tissue samples from normal and lung cancer patients to characterize miR‐96 levels. Quantitative PCR analysis, as shown in Figure 1A, indicated that miR‐96 is expressed at higher levels in lung cancer tissues (*n* = 56) than normal lung tissues (*n* = 19). Further, grouping samples according to the grades demonstrated a trend of prominently increasing miR‐96 level in higher grade lung cancers (Fig. 1B). Accordingly, a higher miR‐96 level was expressed in metastasis‐containing lymph node than non‐metastatic tumours (Fig. 1C).

**Figure 2:**
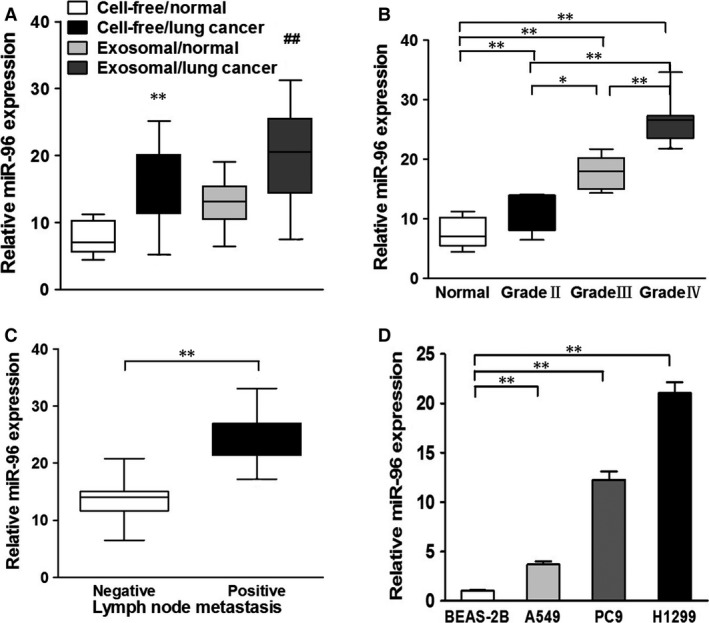
Exosomal miR‐96 expression levels are elevated in the serum of lung cancer patients and in the supernatant of lung cancer cell lines. (**A**) The box plot compares the relative values of cell‐free and exosomal miR‐96 in the serum of cancer‐free (normal, *n* = 19) and lung cancer patients (*n* = 56). **P < 0.01 compared to cell‐free/normal group. ^##^ *P* < 0.01 compared to exosome/normal group. (**B**) Relative expression levels of exosomal miR‐96 in the serum of different stages of cancer patients, *P < 0.05, **P < 0.01. (**C**) Relative expression levels of exosomal miR‐96 in different types of lymph node metastasis. **P < 0.01. (**D**) Relative expression levels of exosomal miR‐96 in the supernatant of different lung cancer cell lines. **P < 0.01. Data were presented as mean ± S.D. Experiments were repeated in triplicate.

Based on the above findings, we went ahead to evaluate whether miR‐96 can be a serum biomarker for lung cancer. In this scenario, we isolated cell‐free serum from lung cancer patients (*n* = 56) and normal patients (*n* = 19) and analysed miR‐96 using qPCR analysis. Our results confirmed the up‐regulation of miR‐96 in lung cancer tissues, as well as in the serum. Considering the role of exosomes as a vehicle for miR‐96, we also analysed exosomal miR‐96 in both lung cancer and normal patients and found that miR‐96 in exosomes was also up‐regulated in cancer samples (Fig. 2A). We also found that exosomal miR‐96 was positively correlated with the cancer grades (Fig. 2B). Lymph nodes with cancer also demonstrated a higher miR‐96 level compared with normal tissues (Fig. 2C). We also compared the exosomal miR‐96 levels in BEAS‐2B, A549, PC9 and H1299 lung cell lines. BEAS‐2B is an immortalized lung epithelial cell line; A549, PC9 and H1299 represent NSCLC cells with increasing invasiveness (A549 < PC9 < H1299) 16, 17, 18. It was found that higher exosomal miR‐96 levels can be seen in cells with higher invasiveness (Fig. 2D), which further validated that exosomal miR‐96 is a biomarker for aggressive lung cancer.

### Down‐regulation of endogenous miR‐96 inhibits the exosome‐enhanced cell proliferation, migration and cisplatin resistance

**Figure 3:**
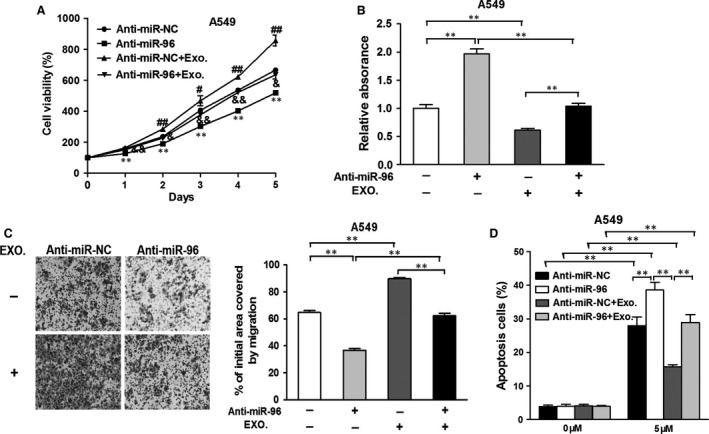
Down‐regulation of miR‐96 inhibits the exosome‐induced cell functions and drug resistance of lung cancer cells. A549 cells were transfected with anti‐miR‐96 inhibitor (anti‐miR‐96) or control antisense RNA (anti‐miR‐NC) and cultured with the exosomes separated from the supernatant of H1299 or not. (**A**) CCK‐8 assay was used to detected cell viability. **P < 0.01 compared to anti‐miR‐96 group. ^#^ *P* < 0.05 and ^##^ *P* < 0.01 compared to anti‐miR‐96 group. &*P* < 0.05 and &&*P* < 0.01 compared to anti‐miR‐NC+exo. group. (**B**) Cells were treated as above, then a sterile 10 μl pipette tip was used to scratch the cells to form a wound when the cell densities were about 90% confluence. **P < 0.01. (**C**) Transwell invasion assay of the cells overexpressing miR‐96 cells with or without LMO7 overexpression. After being fixed, the cells in the bottom of the invasion chamber were measured by the absorbance at 570 nm. **P < 0.01. (**D**) Cells were treated with 5 μM cisplatin or not, and cell apoptosis was analysed by flow cytometry after 48 hrs. **P < 0.01. Data were presented as mean + S.D. Experiments were repeated in triplicate.

To demonstrate the effect of miR‐96 on lung cancer cells, A549 cells were transfected with anti‐miR‐96 inhibitor (anti‐miR‐96), or control antisense RNA (anti‐miR‐NC) and cultured with the exosomes separated from the supernatant of H1299. Treatment with anti‐miR‐96 led to the lowest cell growth rate, and adding exosomes from H1299 promoted the cell growth compared with their anti‐miR‐96 or anti‐miR‐NC counterparts (Fig. 3A). This indicated a tumour‐suppressing function of anti‐miR‐96 and a tumour‐promoting function of exosomes from H1299. This effect can also be reflected in terms of cell migration. While anti‐miR‐96 inhibited cell migratory abilities, adding exosomes from H1299 promoted cell migration (Fig. 3B and C). To confirm the role of miR‐96 and exosomes in drug resistance, cells were treated with cisplatin, followed by analysing cell apoptosis (Fig. 3D). Although cisplatin induced a universally higher apoptosis rate compared with non‐treated groups, adding anti‐miR‐96 resulted in increased apoptosis rate even further, and cells received anti‐miR‐NC and exosomes suffered the lowest apoptotic effect from cisplatin. Collectively, it is obvious that miR‐96 and exosomes play important roles in maintaining the growth, migration and drug resistance of A549 cells. Conversely, anti‐miR‐96 resulted in decreased cell growth, migration and drug resistance. To evaluate the effect of paracrine miR‐96‐carrying exosomes on cell proliferation, we analysed intracellular levels of miR‐96 in A549 cells before and after treatment with H1299‐derived exosomes. As expected, an increase in miR‐96 was seen after exosome treatment (Fig. S2). After depleting H1299‐derived exosomes by incubating A549 cells for 48 hrs after treatment, exosomes from A549‐conditioned medium were isolated and used to treat fresh A549 cells. Interestingly, these exosomes were able to exert similar tumour‐promoting effect as the H1299‐derived exosomes, whereas exosomes from untreated A549 cell did not exert such effect. Transfection with anti‐miR‐96 also suppressed cell proliferation in the same condition (Fig. S3). As another control, we also treated H1299 cells with anti‐miR‐96, which significantly reduced miR‐96 expression (Fig. S4A). On the contrary, anti‐miR‐96 treatment did not down‐regulate miR‐96 expression (Fig. S4B). As a result, exosomes isolated from anti‐miR‐96‐treated H1299 cell did not exert tumour‐promoting effects on A549 cells (Fig. S4C‐F). Taken together, these data indicated that miR‐96‐containing exosomes exhibit a tumour‐promoting role in lung cancer, and down‐regulation of miR‐96 can suppress cell proliferation, migration and drug resistance.

### MiR‐96 directly targets and inhibits LMO7 expression

**Figure 4:**
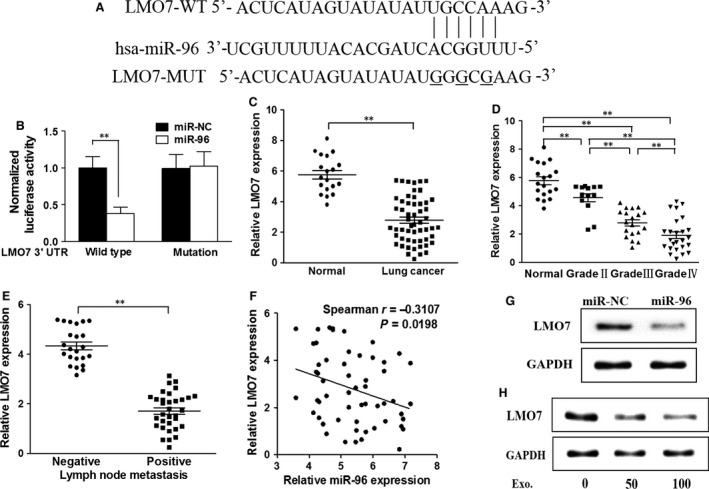
miR‐96 directly targets and inhibits LMO7 expression. (**A**) Putative seed‐matching sites or mutant sites (with underline) between miR‐96 and 3′‐UTR of LMO7. (**B**) Luciferase reporter assay was performed on FaDu to detect the relative luciferase activities of WT and mut LMO7 reporters. Renilla luciferase vector was used as an internal control. **P < 0.01. (**C**) The expression levels of LMO7 in normal tissues and human hypopharyngeal cancer specimens were determined by qRT‐PCR analysis, and the fold changes were obtained from the ratio of LMO7 to GAPDH levels. **P < 0.01. (**D**) Relative expression levels of miR‐96 in different stages of cancer tissues, *P < 0.05, **P < 0.01. (**E**) Relative expression levels of miR‐96 in different types of lymph node metastasis. *P < 0.05. (**F**) Spearman's correlation analysis was used to determine the correlation between the expression levels of LMO7 and miR‐96 in human hypopharyngeal cancer specimens. (**G**) Total proteins of miR‐96‐ and miR‐NC‐expression cells were subjected to Western blotting and detected for LMO7 expression levels. (**H**) A549 cells were cultured with different concentration (0, 50, 100 μg/ml) of exosomes separated from the supernatant of H1299. After 48 hrs, total proteins of each cell was subjected to Western blotting and detected for LMO7 expression levels. Data were presented as mean + S.D. Experiments were repeated in triplicate.

To understand the molecular mechanism of miR‐96 in promoting cancer cell growth, we performed a search of miR‐96 sequence and found that seed‐matching sites exist between miR‐96 and 3′‐UTR of LMO7 (Fig. 4A). These seed‐matching sites are absent in mutant LMO7 gene (LMO7‐MUT). Furthermore, dual‐luciferase reporter assay was carried out to analyse the interaction between miR‐96 and LMO7. As seen in Figure 4B, addition of miR‐96 decreased activity of wild‐type LMO7, but not mutant LMO7, demonstrating the specific interaction between miR‐96 and LMO7. Contrary to the tumour‐promoting role of miR‐96, LMO7 expression was negatively correlated with cancer malignancy. Lower LMO7 expression was seen in lung cancer than normal tissues (Fig. 4C) and relatively lower LMO7 expression was seen in cancer tissue with higher grades (Fig. 4D) and metastatic lymph node tissues (Fig. 4E). Spearman's analysis indicated a negative correlation between LMO7 expression and miR‐96 expression (Fig. 4F). Western blotting analysis also demonstrated that miR‐96 and exosomes from H1299 reduced LMO7 protein expression (Fig. 4G and H).

### Overexpression of LMO7 reverses the stimulating effects of miR‐96

**Figure 5:**
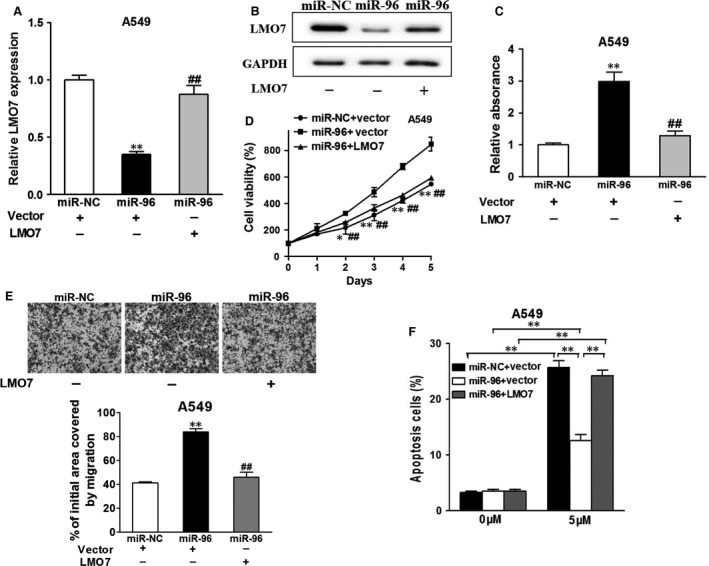
Overexpression of LMO7 reverses the stimulative effects of miR‐96. miR‐96‐ or miR‐NC‐overexpression cells were transfected with vector or LMO7. (**A**,** B**) After 48 hrs, cells were subjected to qRT‐PCR and Western blotting and detected for LOM7 expression levels. (**C**) CCK‐8 assay was used to detected cell viability. *P < 0.05 and **P < 0.01 compared to miR‐NC+vector group. ^##^ *P* < 0.01 compared to miR‐96 + vector group. (**D**) Cells were treated as above, then a sterile 10 μl pipette tip was used to scratch the cells to form a wound when the cell densities were about 90% confluence. **P < 0.01, ^#^ *P* < 0.05, ^##^ *P* < 0.01. (**E**) Transwell invasion assay of the cells overexpressing miR‐96 cells with or without LMO7 overexpression. After being fixed, the cells in the bottom of the invasion chamber were measured by the absorbance at 570 nm. **P < 0.01. ^##^ *P* < 0.01. (**F**) Cell were treated with 5 μM cisplatin or not, and cell apoptosis was analysed by flow cytometry after 48 hrs. **P < 0.01 indicates significant difference. Data were presented as mean + S.D. Experiments were repeated in triplicate.

As miR‐96 targets LMO7 to promote cancer proliferation, migration and drug resistance, we continued to investigate whether overexpression of LMO7 was able to reverse the stimulating effects of miR‐96. As shown in Figure 5A and B, transfection of LMO7 plasmid reversed effect of miR‐96 in down‐regulating LMO7 expression in both mRNA and protein levels, indicating the direct interaction between LMO7 and miR‐96. Expectedly, while miR‐96 promoted cell growth, overexpression of LMO7 nearly brought cell growth rate to its original level (Fig. 5C). Similarly, LMO7 overexpression also inhibited cell migration (Fig. 5D and E) and compromised the heightened drug resistance rendered by miR‐96 (Fig. 5F).

## Discussions

In the present study, we analysed miR‐96 expression in tissue, serum and isolated exosomes from normal or lung cancer patients and verified that, apart from elevated miR‐96 expression in lung cancer in the tissue level, a positive correlation existed between cell‐free/exosomal miR‐96 level in serum and lung cancer tissues. We paid special attention to miR‐96 carrying exosomes as miR‐96 are enriched in exosomes, which serve as effective cancer regulators. Importantly, exosomal miR‐96 level was demonstrated to be useful in delineating lung cancer stages due to the increasing expression of miR‐96 in higher‐stage cancers. Lymph nodes that contain metastatic tumour also showed up‐regulated miR‐96 expression. To the best of our knowledge, our study is the first to report the diagnostic and prognostic value of exosomal miR‐96. Being actively secreted by cells, circulating exosomes were discovered in almost all bodily fluids, such as blood cerebrospinal fluid, saliva, milk and urine. Diagnostic tests that involve non‐invasive procedures such as simple blood draw are highly desirable and patient friendly. The increasing level of exosomal miR‐96 in serum could be used to detect lung cancer early, determine lung cancer aggressiveness accurately and predict patient survival. This serum‐based approach is advantageous than biopsy, an invasive procedure that serves as the main tool for lung cancer risk assessment. In addition, this serum‐based miR‐96 biomarker may even serve as an alternative to other imaging modalities, such as computed tomography and magnetic resonance imaging, the clinical utility of which is impaired by their high cost and ineffectiveness to determine lung cancer risk 19, 20, 21, 22. Our results concur with previous studies showing that other miRNA families, such as the miR‐200 family, within circulating exosomes possess both diagnostic and prognostic relevance in patients with epithelial ovarian cancer 23. Considering the lack of miRNAs as serum biomarker for lung cancer diagnosis, the establishment of exosomal miR‐96 as a lung cancer biomarker may potentially lead to more accurate and efficient clinical management of lung cancer.

Another issue that hinders effective lung cancer treatment is frequent drug resistance and subsequent recurrence in lung cancer patients. Cisplatin treatment, as a platinum based therapy, is part of the first‐line treatment for lung cancer 24. It acts by causing DNA damage to cancer cells, which activates apoptosis. However, while 20–40% patients with metastatic lung cancer experience partial response to cisplatin, most responders develop cisplatin resistance and show relapses 25. This eventually results in a dismal 5‐year survival rate of 15–25% 26. Factors that likely contributed to the failure include our inability to predict individual patients with potential drug resistance, as well as the lack of knowledge in how lung cancer acquires drug resistance. While a number of genetic pathways have been proposed to contribute to cisplatin resistance, there is still an ongoing need to pinpoint the exact mechanism involved 27, 28, 29. As mRNAs play a direct role in cisplatin resistance, miRNA as a broad regulator of mRNAs may potentially help unravelling the mechanism of cisplatin resistance. In this study, we showed that, concomitant with decrease in cell viability and migration, anti‐miR‐96 transfection also reduced cisplatin resistance in A549 cells. On the contrary, exosomes from H1299 cells, which contained miR‐96, significantly increased cisplatin resistance. Therefore, miR‐96 level may be a strong indicator of lung cancer cisplatin resistance. Further development of exosomal miR‐96 in serum as a predictor of cisplatin resistance could improve treatment planning, for instance, by determining timing to provide alternative drugs, or combining radiotherapy. More importantly, the enhanced apoptotic effect associated with anti‐miR‐96 transfection provided us with a viable option to overcome developed cisplatin resistance. New advances in gene therapy, such as potential delivery vehicles for miRNAs 30, 31, 32, 33, could facilitate development of anti‐miR‐96 therapeutic strategies. This would have tremendous impact in decreasing lung cancer mortality rates and providing timely therapeutic interventions and effective disease management.

Besides, we also confirmed LMO7 gene as a potential target of miR‐96. Contrary to the tumour‐promoting role of miR‐96, LMO7 is a tumour suppressor of lung cancer, as demonstrated in our qPCR analysis that showed a decreased level of LMO7 in lung cancer patients. A decreasing level of LMO7 was also seen in high‐grade lung cancer and lymph node with metastatic tumours. This is consistent with previous results that low LMO7 level was correlated with a poor prognosis of lung cancer patients 34. LMO7 possesses seed‐matching sites with miR‐96 at 3′‐UTR, and we showed that LMO7 overexpression and miR‐96 counteracted each other in lung cancer. LMO7 overexpression down‐regulated miR‐96 level, and miR‐96 transfection down‐regulated LMO7 expression. This translated to the fact that the promoting effect of miR‐96 transfection was compromised by LMO7 overexpression. Indeed, a negative correlation was found between miR‐96 and LMO7 as revealed by Spearman's analysis. Unsurprisingly, this counteracting effect can be manifested in the role of both miR‐96 and LMO7 in regulating cisplatin resistance. Overexpressing LMO7 compromised the cisplatin resistance from miR‐96 transfection. Due to this reason, LMO7 could be another therapeutic target that directly regulates miR‐96′s tumour‐promoting function.

## Conclusions

We have identified exosomal miR‐96 as a serum biomarker of malignant lung cancer. MiR‐96 regulates lung cancer progression by targeting LMO7. New diagnostic or therapeutic strategies could be developed by targeting the miR‐96‐LMO7 axis.

## Conflicts of interest

There are no conflicts of interest.

## Supporting information


**Figure S1** Identification of exosomes.
**Figure S2** The miR‐96 expression level of A549 is up‐regulated by the exosomes from A549.
**Figure S3** Downregulation of miR‐96 inhibits the exosome induced cell proliferation.
**Figure S4** Silence of endogenous miR‐96 revises the exosomal gain of function.
