# Supplementary material for: Circulating exosomal microRNA‐96 promotes cell proliferation, migration and drug resistance by targeting LMO7
Source: J Cell Mol Med. 2016 Dec 27;21(6):1228–36. doi: 10.1111/jcmm.13056 (PMC5431139; doi:10.1111/jcmm.13056)
Supplement: Supplementary file 1 — Figure S1 Identification of exosomes. Figure S2 The miR‐96 expression level of A549 is up‐regulated by the exosomes from A549. Figure S3 Downregulation of miR‐96 inhibits the exosome induced cell proliferation. Figure S4 Silence of endogenous miR‐96 revises the exosomal gain of function. [file JCMM-21-1228-s001.docx]

**
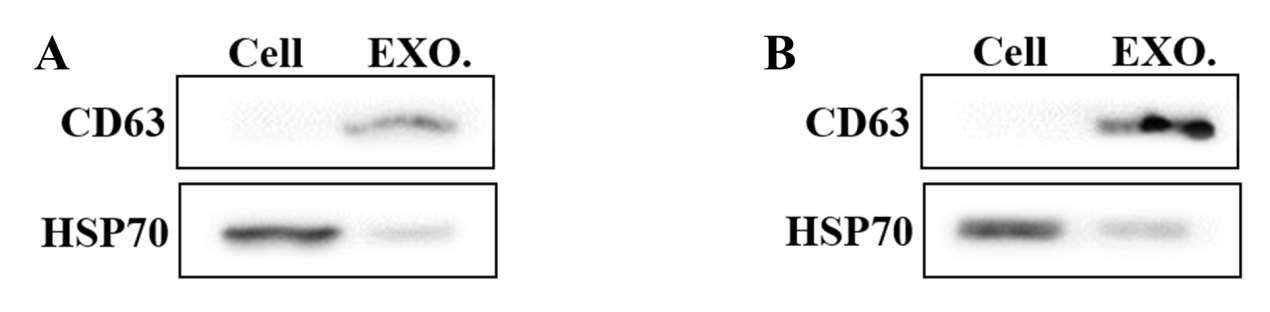
**

**Fig. S1. Identification of exosomes.** (A) Exosomes were isolated by ExoQuick protocol and were subjected to western blotting to analyze CD63 and HSP70 expression levels. (B) Exosomes were isolated by ultracentrifugation protocol and were subjected to western blotting to analyze CD63 and HSP70 expression levels.


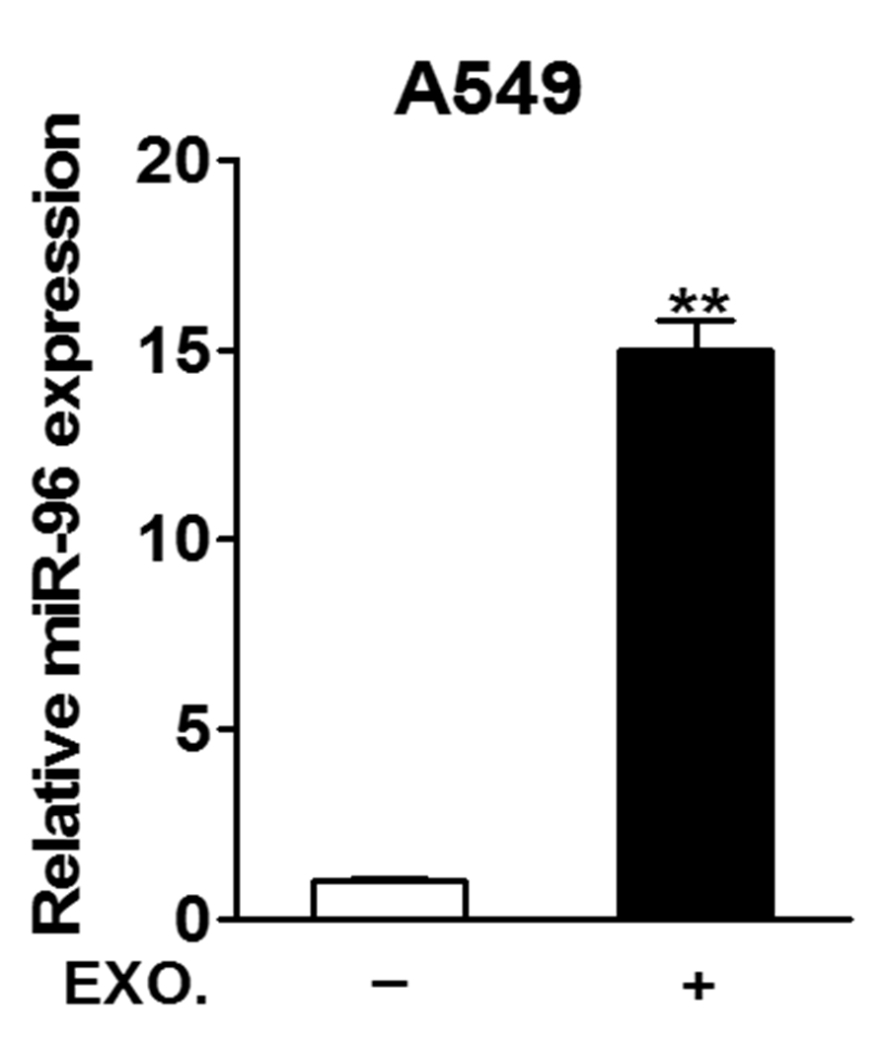


**Fig. S2. The miR-96 expression level of A549 is up-regulated by the exosomes from A549.** The A549 cell line was cultured with the exosomes from the supernatant of H1299. After 48 h, cells were subjected to qRT-PCR and detected for miR-96 expression levels. **p<0.01.

**
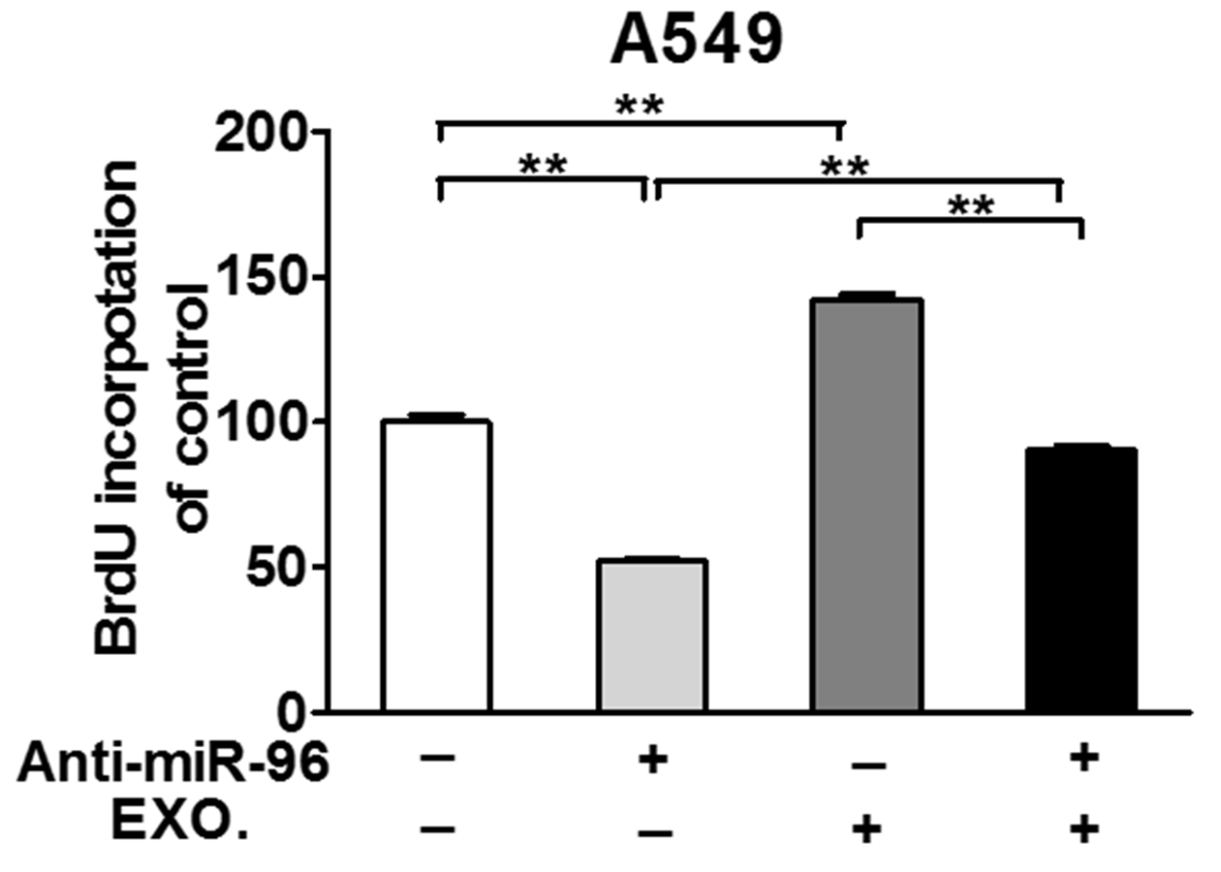
**

**Fig. S3. Downregulation of miR-96 inhibits the exosome induced cell proliferation.** Cells were treated as same as that in Fig. 3. BrdU assay was employed to analyze the proliferation of the cells.

**
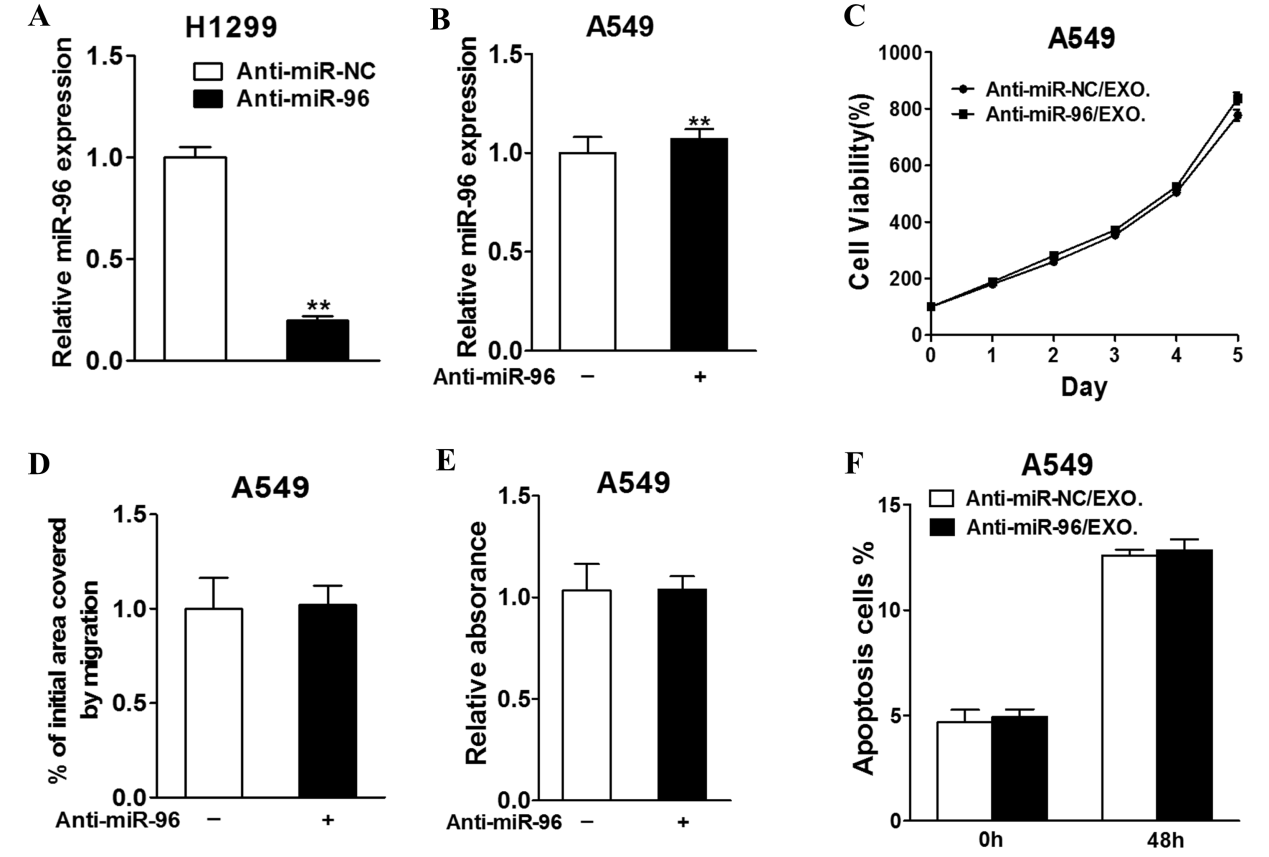
**

**Fig. S4. Silence of endogenous miR-96 revises the exosomal gain of function.** H1299 cells were transfected with Anti-miR-96 or Anti-miR-NC. (A) After 48 h, cells were subjected to qRT-PCR and detected for miR-96 expression levels. (B) Exosomes were isolated from supernatant of the cells above and added to the medium of A549. After 48 h, cells were subjected to qRT-PCR and detected for miR-96 expression levels. (C) CCK-8 assay was used to detected cell viability. **p<0.01. (D) Cells were treated as above, then a sterile 10 μl pipette tip was used to scratch the cells to form a wound when the cell densities were about 90% confluence. **p<0.01. (E) Transwell invasion assay of the cells. After being fixed, the cells in the bottom of the invasion chamber were measured by the absorbance at 570 nm. **p<0.01. (F) Cell were treated with 5 μM cisplatin or not, and cell apoptosis was analyzed by flow cytometry after 48h. **p<0.01.
